# Data Work: How Energy Advisors and Clients Make IoT Data Accountable

**DOI:** 10.1007/s10606-017-9293-x

**Published:** 2017-06-23

**Authors:** Joel E. Fischer, Andy Crabtree, James A. Colley, Tom Rodden, Enrico Costanza

**Affiliations:** 1 Mixed Reality Laboratory, School of Computer Science, University of Nottingham, Nottingham, UK; 2 UCLIC, University College London, London, UK

**Keywords:** Internet of things, Ethnomethodology, CSCW, Collaborative work

## Abstract

We present fieldwork findings from the deployment of an interactive sensing system that supports the work of energy advisors who give face-to-face advice to low-income households in the UK. We focus on how the system and the data it produced are articulated in the interactions between professional energy advisors and their clients, and how they collaboratively anticipate, rehearse, and perform data work. In addition to documenting how the system was appropriated in advisory work, we elaborate the ‘overhead cost’ of building collaborative action into connected devices and sensing systems, and the commensurate need to support discrete workflows and accountability systems to enable the methodical incorporation of the IoT into collaborative action. We contribute an elaboration of the social, collaborative methods of data work relevant to those who seek to design and study collaborative IoT systems.

## Introduction

The Internet of Things (IoT) has been portrayed as a key enabler of a “second digital revolution” in sectors such as transport, energy, healthcare, agriculture, cities and buildings (Walport 2014). One of the promises is that the IoT will empower individuals “to make better decisions” about energy consumption (FTC 2015); a process in which access to, and use of IoT *data* is seen as indispensable. However, an understanding of *data work,* i.e., the social practices *in and through* which IoT data is accountably collected, used, and acted upon is arguably underdeveloped, as are the resulting implications for the design of interactive IoT systems. In turn, this paper introduces and further develops a systematic understanding of *data work,* by building on, and extending prior research unpacking the ways in which energy advisors employed by a UK-based charity exploit IoT data to support their professional advice-giving practices (Fischer et al. 2014, 2016).

The starting point for our work was a study of the professional work practices involved in tailoring energy advice to particular households (Fischer et al. 2014). The initial understanding from this study informed the design of a non-interactive ‘seed prototype’ – a connected data collection kit – which was trialled to explore the ways in which sensed data including temperature, humidity, electricity and natural gas consumption data might be leveraged to deliver and tailor energy advice (Fischer et al. 2016). This further field study made it visible that sensor data is indexical to the sites and practices of its production (Garfinkel 1967), and that the ‘facticity’ of the data therefore had to be ‘articulated’ (Schmidt and Bannon 1992) in situ between advisor and client. We previously described this accomplishment as *data work*, and detailed the methodical ways in which it is accomplished in advisor-client interaction (Fischer et al. 2016).

This paper builds on these prior efforts a) by extending the range of ‘data sources’ – i.e., Internet-enabled sensing – made available to energy advisors, and b) by introducing an interactive system that makes the data produced by the extended sensor kit available for visual interrogation and annotation. This extended system called ‘CharIoT’ was deployed in 10 households in the UK. We conducted observations of in-home visits when the sensing kit was installed in clients’ homes, workshops between advisors as they tried to work through and make sense of the resulting data, and subsequent advice visits in which the data was leveraged to support clients. As in our previous study we focus here on unpacking how the data was articulated in practice, particularly on novel methods of data work that emerged in articulating a much richer data set.

The practical aims of the research project, as noted above, were to enhance professional practice through design and thus enable energy advisors to offer more detailed advice to their clients. While advisors have become well versed in the doing of data work, clients only experience data work on a single occasion, so expertise lies with the advisors to some large extent. We do not consider the CharIoT system from the individual perspective of clients then, but rather examine how clients were practically implicated in the doing of data work. Thus, the paper examines the collaborative interactional performance of data work, including the working practices or methods in and through which IoT data is accountably anticipated during installation, is rehearsed with colleagues prior to advice-giving home visits, and its meaning negotiated in situ with clients. Our findings subsequently elaborate organising features of data work. In discussion we consider how these might be supported more generally by building discrete workflows and ‘accountability systems’ (Strauss 1985) into interactive IoT technologies to enable their *methodical* incorporation into collaborative action (Button et al. 2015). Our contribution thus lies in elaborating the ***collaborative methods of data work*** and the implications these have for the design of interactive IoT technologies more generally, including accounting for data capture and use, situating sensors, capturing contextual metadata (i.e., data *about* data), making sense of the data, and turning data into action.

## Related work

Our work is located at the intersection of energy-related research, work-practice studies, and a concern with articulation work in CSCW. We briefly review relevant literature and highlight its relationship to the work presented in this paper, before moving on to describe our efforts to support the professional practice of energy advisors through design.

### Energy, sustainability and sensor data

Our work stands apart from the mainstream of energy-related work in HCI, which has largely focused on Energy Consumption Feedback (ECF) to raise awareness and encourage people to change their behaviour (DiSalvo et al. 2010; Pierce and Paulos 2012). ECF has been criticised for ignoring the situated practices in which energy consumption is embedded (Strengers 2011), and this provides the premise to ‘go and look’ at such practices that motivates our own work. It resonates too with recent work that has called for “opportunities to study people’s practices that include the everyday use of IoT technologies” (Robertson and Wagner 2015). Coupled to this, advances in computing and sensor technology have led to the development of novel applications that enable, for example, the measurement of air quality (Jiang et al. 2011; Kim and Paulos 2010), occupancy (Scott et al. 2011), and CO2 data (Jacobs et al. 2013). The same advances have enabled us to prototype our own interactive sensor-based system. A common research focus has also been the design and evaluation of interactive systems to help make sense of energy data. In our design, we have been inspired by prior work that has studied visualisations, annotations and other means to inspect and make sense of sensor data (Costanza et al. 2012).

Less prevalent, but equally as informative is research that pays attention to collaborative energy-related practices. Dillahunt’s studies of low-income rented properties has shed light on social issues that prevent energy-related improvements, for example, such as lack of control and ownership (Dillahunt et al. 2009), conflicts between landlords and tenants (Dillahunt et al. 2010), and lack of connectedness in the community (Dillahunt and Mankoff 2014). Studying a workplace setting, Bedwell et al. (2016) have also highlighted the ways in which employees collaboratively manage energy consumption. Our work is also informed by and speaks to research concerned with the relationship between sensor data and its intelligibility in a domestic context (e.g., Chetty et al. 2010; Dong et al. 2015; Pousman et al. 2008). Complementing this, Human-Data Interaction has recently been proposed as an emerging field that acknowledges “the inherently social and relational character of data” (Crabtree and Mortier 2015). Our fieldwork pays attention to the various forms of situated and practical reasoning that people collaboratively apply when articulating sensor data, such as reasoning about place, time, people, practices and events (Tolmie et al. 2016).

### Work-practice

Our research has a particular focus on work-practice (Button and Harper 1995) and draws on fieldwork to inform and shape systems design. Work-practice studies are traditionally associated with the workplace and paid labour. However, as Crabtree et al. (2009) point out, work-practice is a generic feature of human interaction and collaboration wherever it occurs. Work-practice spans and blurs traditional boundaries and lends itself well to the study of advisors’ work, which takes place not only in offices but in clients’ homes, and is not-for-profit in nature. Related work in non-profit workplace settings has, for example, examined information management (Merkel et al. 2007), coordination and awareness (Stoll et al. 2010), participatory design with community groups (Merkel et al. 2004), fundraising (Goecks et al. 2008), and volunteer coordination (Voida et al. 2012).

In this paper we draw on previous ethnomethodological studies of the work-practices implicated in the conduct of not-for-profit energy advice-giving work (Fischer et al. 2014, 2016) to shape the design of an IoT system that supports interaction and collaboration between energy advisors and their clients. The system has been extended, redeployed in client’s homes, and subject to further field study, the results of which are presented in this paper. In this respect our work is also related to technology deployments in the home which, as Tolmie and Crabtree (2008) point out, is often oriented to by household members as something done *to* them rather than done *for* them. Our work seeks to exploit technology to deliver beneficial outcomes *for* clients through supporting professional practice. In doing so it trades on and further unpacks the inherently collaborative character of the work that advice-giving turns upon.

### Articulation work

The collaborative work of energy advice-giving turns upon the ‘articulation’ of sensor data (Fischer et al. 2016). Articulation work is foundational to CSCW and has its origins, as Schmidt and Bannon (1992) note, in sociology and the interactionist studies of work done by Anselm Strauss (1985). Strauss recognised that collaborative action involves ‘a supra type of work’, which Schmidt and Bannon characterised as the ‘overhead cost’ of collaboration. The overhead cost consists of making work or action in the round accountable to participants. Without this it is impossible for actors to ‘mesh’ their actions together and thus pull off the collaborative endeavour they are engaged in. Importantly, as Strauss made perspicuous, this is often provided for through the construction of ‘accountability systems’. An accountability system may be a simple paper form or a complex computational system. Whatever the case, articulation work orients us to understanding how the collaborative action and interaction implicated in technology use is made accountable to the parties involved in doing it, which in turn provides insights for the development of collaborative systems. Our work thus seeks to understand how the IoT is articulated and made accountable in the interactions between advisors and their clients.

## Designing to support energy advice

This section details the co-design process shaping the CharIoT system^1^, i.e., the new IoT sensor system reported in this paper. It provides relevant background on the energy advisors and the kinds of households they typically visit, prior ethnographic and design work, and a detailed description of the system itself.

### Energy advice and in-home visits

The energy advisors involved in our research are employees of the Centre for Sustainable Energy (CSE), a not-for-profit charity based in Bristol, UK. Practically, the project aimed at leveraging the IoT to support and enhance the provision of in-home energy advice to their clients. In-home visits involve the most vulnerable of CSE’s clients and include sick children, the elderly, disabled, and infirm. This cohort is routinely affected by compound issues to do with education, employment, personal finances, health and bodily ability. The winter months can be particularly problematic, when the proportion of (already low) income needed to be spent on fuel to keep warm rises. To complicate matters, vulnerable people often live in rented housing in poor condition, which they lack the funds to improve. The energy advisors’ work involves diagnosing the causes of high bills and health risks (e.g., damp and mould), recommending material and behavioural improvements, and reporting to third parties to make the case for improvements on their client’s behalf (e.g. landlords, councils, and energy suppliers).

### Formative ethnographic findings

An earlier ethnographic study of advisors’ work practices (Fischer et al. 2014) informed the development of an initial ‘seed prototype’, a previous version of the IoT system presented in this paper. The purpose of the study was to identify opportunities for technology support, in particular for *sensors* to be installed in client’s homes and *digital representations* of sensor data to be provided to the advisors to help identify the causes of problems and improve their ability to tailor advice to clients. With respect to sensors, it was found that electricity and natural gas sensors would be insufficient for these purposes. Sensing issues such as dampness, mould, and cold require ambient environmental data, such as temperature and humidity sensing. For example, low temperatures and high levels of humidity may lead to mould growth and may affect health.

Prior work also showed the need for graphical visualisations that advisors could show to clients. Simple line charts were deemed the preferred type in design workshops with advisors. In addition, prior findings suggested that CSE’s clients have lower access to broadband and digital devices, in line with statistics that show that 42% of low-income households in the UK do not use the Internet (Dutton and Blank 2013). As a result, we initially experimented with a self-contained (3G mobile network-based) infrastructure. However, this proved unreliable and we opted to make broadband access a requirement for participation in the study following advice from CSE that they are seeing more and more client households equipped with broadband.

The initial seed prototype allowed us to place a simple sensor kit, which packaged a temperature, humidity and light sensor in a single networked device - in client’s homes and to furnish advisors with data ‘print outs’ prior to in-home visits. This enabled the advisors to develop an initial understanding of the client’s problems and their potential causes, and to identify energy-related issues and topics for discussion during in-home visits. Ethnographic study of the seed prototype in use provided detailed insight into the collaborative work involved in articulating the data visualisations generated by the sensor kit. This ‘data work’ involved advisors and clients working together to ‘unpack’ the indexical character of simple line charts – i.e., making data visualisations accountable to local activities and events. In turn, this work provided the basis for advisor and client to formulate situationally appropriate remedial actions (Fischer et al. 2016).

Deployment and study of the seed prototype gave rise to a number of new design requirements: a) the system should support multiple temperature and humidity sensors, and enable data collection from multiple rooms; b) the system should incorporate outdoor temperature in order to disambiguate indoor temperature fluctuations, and CO2 in order to disambiguate occupancy; c) the system should enable flexible data visualisation across multiple data sources, including filtering, highlighting, and zooming in on interesting periods and sections of the data; d) the system should convert raw electricity and gas measurements for selected periods of time into monetary values; e) the system should allow advisors to annotate the data in order to support pre-home visit ‘rehearsal’ of the data and in situ ‘performance’ of data-driven advice.

### Co-designing the CharIoT system

In response to the requirements that emerged from prior work (Fischer et al. 2016), researchers and advisors undertook a co-design process to create an interactive IoT system through a series of iterative prototyping workshops. This process began with a half-day design workshop, in which the requirements gathered from previous work were shaped into more concrete ideas for developing a working interactive IoT system. This was followed by the development of wireframe interface ‘walkthroughs’ on paper, which were developed by the researchers and presented to the energy advisors as part of a second half-day workshop.

**Figure 1:**
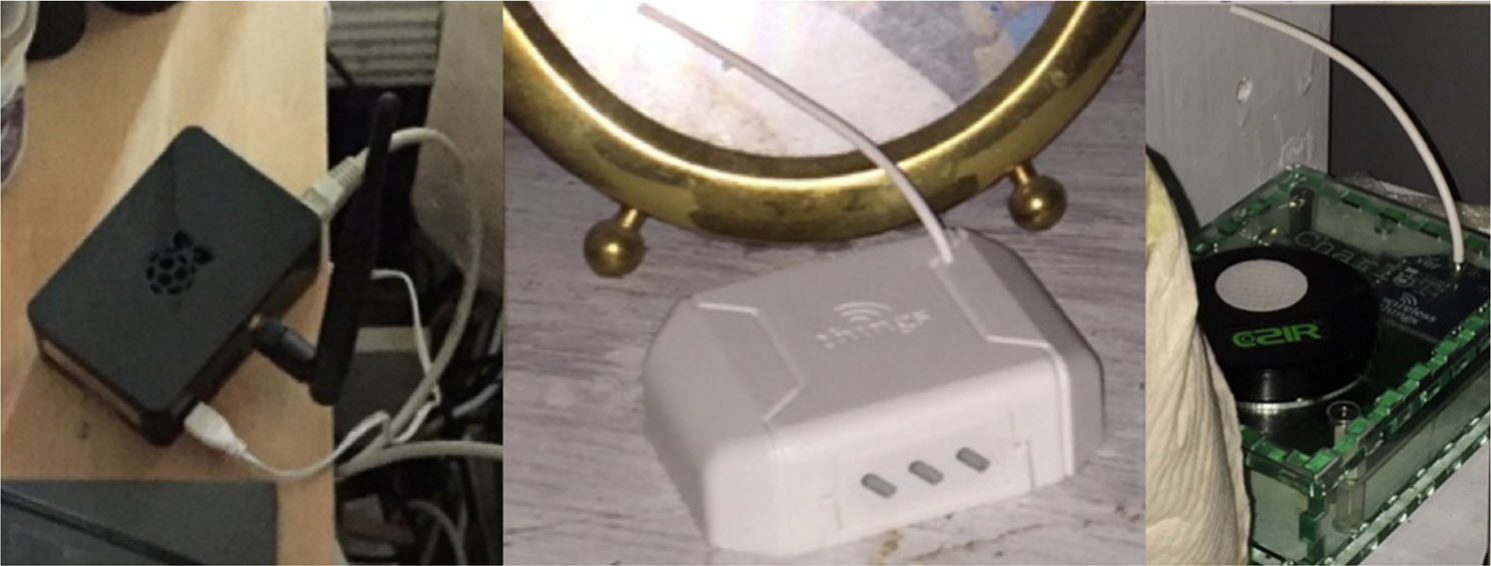
Hub (*left*), temperature/humidity sensor (*middle*) CO2 sensor (*right*) deployed in situ.

The second workshop led to a more detailed critique by energy advisors of the specific functionality of the system, as they began to imagine making use of the proposed interactive IoT system as part of their existing energy advice-giving practices. The refined requirements gathered from analysis of data from the second workshop informed the development of a second set of wireframe prototypes for the interactive system, which would allow energy advisors to organise, deploy and monitor multiple sensors across multiple homes. These wireframes formed the basis of a third half-day workshop to critically analyse the way in which advisors might interact with sensors and sensor data as part of the energy advice process. The outcomes of the final design workshop were used to refine the system specification and resulted in CharIoT, an extend IoT system consisting of the following *sensor kit* (Figure 1):

- A Raspberry Pi based hub, which connects to the client’s broadband via Ethernet and receives radio signals from the wireless sensors and relays data to an online system.
- Off-the-shelf, battery-powered wireless sensors to monitor temperature and humidity.
- Custom-built, battery-powered wireless sensors to monitor temperature, humidity, and CO2.

**Figure 2:**
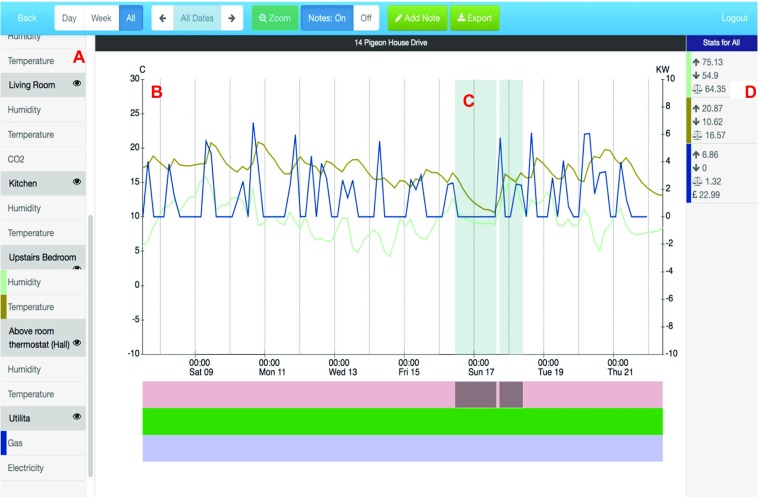
The interactive web app enables (**a)** filtering (show/hide) of sensor data sources, (**b)** pan and zoom time series line charts, (**c)** annotations (highlights) viewable on click, and (**d)** stats for selected sources (min/max, average, cost in £).

In addition to the sensor kit, the CharIoT system also provides an *interactive web app*, which includes:

- A configuration utility, which allowed advisors to assign sensors to households and add basic information about households to the system.
- A dashboard, which provides an overview of all sensor kit deployments and shows the most recent readings and battery levels for all sensors.
- A data viewing tool (Figure 2), which was designed to enable advisors to view and annotate sensor data, and to support articulation of the data between advisors and clients.

This beta-level system was then given to energy advisors to deploy in their own homes for testing, allowing them to get a feel of what it is like to have real data recorded and visualised. The workshop following this beta testing focused on understanding the ways in which advisors accounted for the data recorded in their homes and what issues if any they anticipated would arise as part of a live system deployment with real clients in the wild. Further refinements made to the system as a result of the beta testing included room-level filtering, PDF export, visual improvements, and general bug fixing. A final system was given to the advisors along with a training session on how to set up and deploy sensors.

## In-the-wild deployment

The CharIoT system was deployed in 10 UK homes in the Bristol area for three to four weeks over the winter months of 2015/16 after approval from our University’s ethics committee. The deployment furnishes us with a field site to study the situated, collaborative accomplishment of data work. This section briefly describes the advisors’ role, the participants in our study, the structure of the deployments, and data collection and analysis.

### Advisors’ role

In this deployment we worked closely with four advisors. While their main responsibility continued to be providing advice during in-home visits, their role also involved managing the end-to-end process of deployments, which included three home visits and office work. Tasks included recruiting participants, installing the sensor kit, visually inspecting and annotating the sensor data using the interactive system, providing advice to clients drawing on the data, and wrapping up the deployments. The three advisors split up the 10 deployments opportunistically (by availability) between them, but generally attempted to look after each case for the whole duration of the deployment.

### Participants

Ten homes were recruited to take part in our study after ensuring participants met CSE’s recruitment criteria. Two households subsequently dropped out. Out of the eight remaining households, three households had people over 70 years of age, four had children below the age of five, all eight were low-income households (less than £16,000 per year, compared to the median national household income of £26,000), six reported their homes were colder than they preferred in winter, three reported problems with damp and mould, five reported they struggle or sometimes struggled to pay their fuel bills, and four homes had people with illnesses made worse by the cold. The latest UK government statistic report that 2.38 million households were in fuel poverty in 2014 (DECC 2016).

### Home visits and workshops

Deployment, use and study of the system was facilitated by the advisors through a sequence of three home visits. First, advisors conducted an *installation visit* to place the sensors in the participant’s home (one sensor was typically placed outside, and up to four were distributed in different rooms within the home). After about two weeks, an *advice visit* was done in which advisors drew on the interactive system to work through perceived problems together with clients. After a further one to two weeks, the sensor kit was collected in a *final visit,* and the participants were asked about their experience and whether they did anything differently as a result. In addition, advisors and researchers conducted two workshops. In order to prepare for the advice visit, a *rehearsal workshop* was conducted, where the data from homes were reviewed. Further, a *debrief workshop* was held at the end to reflect on the deployments.

### Data collection and analysis

We treated the deployments as opportunities and subject matter for fieldwork. To capture the collaborative work involved in doing in-home visits, a fieldworker accompanied the advisor and recorded their interactions with clients. Data captured includes about 20 hours of audio and video material, along with fieldnotes. The audio data was transcribed and analysed, taking into account the fieldnotes. Our analytic orientation is ethnomethodological and seeks to identify the methodical ways in which the members of a setting naturally and accountably order their activities in interaction (Garfinkel 1991). Two experienced fieldworkers worked through the transcripts using the ‘horizontal/vertical slicing’ technique (Crabtree et al. 2012) to surface the sequential organisation of interaction and drill down into the methodical ways in which the interactive IoT system was articulated in practice by those who were party to its use.

## Understanding data work

We adopt the *data work framework* developed in previous work to explicate the methodical ways in which the energy data furnished by the new interactive IoT system was articulated in the interactions between advisors and their clients (Fischer et al. 2016). To briefly recap, this framework orients us to discrete phases of data work, including ‘anticipation’, i.e., the articulation work that occurs during installation; ‘rehearsal’, i.e., the articulation work that occurs before an in-home visit; and ‘performance’, i.e., the articulation work that occurs during in-home visits. However, setting aside these particular phases observable in our case we would contest that ‘data work’ understood more broadly is indeed a necessary feature of any efforts to make sense of and exploit data; it is merely the specificity of the setting we have studied that makes it perspicuous in the particular guises that the following sections unpack (cf., Bannon et al. 1993). We elaborate key findings by offering conversational extracts or ‘vignettes’ from the field studies. To provide topical continuity, most vignettes relate to the same case (one household). Vignette 1–4 have been taken from the installation visit, in V5–7 advisors discuss the same household’s data in a workshop, and V10 is taken from the advice visit. V7–8 relate to a different household. It is worth noting that despite necessarily elaborating specific cases, there is nothing special about the selected vignettes; we have selected them as exemplars of the ordinary, routine doing of data work running through our data corpus. The abbreviations used to refer to the speakers are advisor (A), client (C), and researcher (R) who accompanied advisors on in-home visits to gather data and help install the sensor kit. Numbers are added if more than one of these is party to the interaction.

### Anticipation

Anticipating data is the first job of work involved in making the technology work in situ*.* It is done by administering a questionnaire during an in-home interview with the client. In the first deployment (Fischer et al. 2016), the questionnaire was used to *profile* the property (e.g., fuel type, heating system, appliances), the occupants (e.g., number, type and age of people living in the home), their everyday routines (e.g., how they use the heating system, dry their clothes, ventilate the home, etc.), and to establish the client’s main concerns (e.g., damp and mould, high bills, cold home, etc.). This ‘contextual data’ helped the advisors make subsequent sense of the data produced by the sensor kit.

As a result of the first deployment, advisors amended the questionnaire to gather *more* contextual data in order to *better understand* the indexical character of sensor data when rehearsing for the advice visit. Thus, a new section was added to the questionnaire to capture property information (age, type and wall type), energy efficiency measures, and specific issues with condensation, damp, mould and draught. It was further amended to capture more detail about the occupants, particularly health conditions exacerbated by cold and damp, such as asthma, and whether or not anyone relied on any electrical medical equipment. The questionnaire was also amended to capture not the just the type of heating system and the make and model of the boiler and thermostat, but also the settings used by the client, whether or not they used a programmable timer and if so how they used it, and whether they were using any secondary heating sources such as electric heaters. Further detail was also captured about the occupants’ cooking habits (e.g., how often they typically used the hob, oven, and kettle), and a checklist to capture any frequently used electrical equipment. The locations sensors were placed in were also noted for future reference.

The questionnaire is an ‘accountability system’. As the following vignettes illustrate, it is not simply used by the advisors to elicit contextual data - not simply a matter of asking questions and noting down responses. Rather, the questionnaire is used methodically to make the IoT system accountable to clients: to articulate reasons for placing the IoT system in the client’s home, and to articulate what the sensors are, the data they gather, what purposes it will be used for, etc. Seen and treated as an accountability system, the questionnaire thus allows advisor and client to mesh their actions together and collaboratively *introduce* the technology into the home, *situate* it, and project its subsequent *use*. The following vignette illustrates how in the course of administering the questionnaire the IoT system gets introduced into the home.

- **Vignette 1. Introducing sensors into the home**
- Introducing sensors into the home
- A: Okay, so I think they [previously] asked about whether you find that you’re cold? That your home is colder than you’d like it to be sometimes in winter?
- C: Yes.
- A: And that there were some difficulties with damp and mould?
- C: Yes, in our bedroom.
- A: Is it mainly the bedroom or is it in other rooms?
- C: No, it’s just our bedroom.
- A: Just the bedroom, OK.
- C: And like in the window and all over the ceiling.
- A: OK. We can put the sensors in. They’re very good for seeing how humid the room gets and the temperature, and indicate whether it can lead to damp and mould. So we can kind of test that in the room for you?
- C: Yes.

As the above vignette makes visible, the introduction of sensors into the home is ‘occasioned’ (Zimmerman and Pollner 1970), and occasioned in a number of ways that make the introduction accountably reasonable. Thus, we can see that the current interaction between the advisors and client is occasioned by prior contact between the client and CSE, which warrants the advisors being in the client’s home ‘here and now’; the warrant being that “your home is colder than you’d like it to be” and that there are “some difficulties with damp and mould”. It is this warrant that occasions the elicitation of contextual data and results in the articulation of a specific problem “in our bedroom”. This, in turn, occasions the proposal to “put the sensors in” and “test” how “humid the room gets and the temperature”, both of which “can lead to damp and mould”. In home after home we see the same methodical procedures at work in the articulation of contextual data and the occasioning of warrants, problems, and proposals making the introduction of the sensing kit an accountably reasonable thing to do.

We also see how elicitation of contextual data enables advisors and clients to collaboratively situate sensors in home after home. Thus, as the following vignette makes visible, occasioning the introduction of the technology into the home enables advisors and clients to work out just where to situate sensors in the home.

- **Vignette 2. Situating sensors in the home**
- Situating sensors in the home
- Client leading advisor and researcher upstairs to bedroom; advisor continues to elicit contextual data:
- C: [Enters bedroom] Excuse the mess.
- A: You can smell the - kind of mouldy - the dampness.
- C: All the clothes around are ready to move because we can’t put them in the cupboard anymore; because it’s all just like that in cupboard
- R: Oh gosh! OK.
- C: So excuse (…)
- A: Is it alright if I take a photo of (…)
- C: Yes. You can see it’s quite bad in here.
- A: Have you tried wiping it down at all?
- C: Yes. I quite constantly keep wiping it. The window is normally open as well, but the house is cold to keep the window open. I don’t know if you want to put it up on top of the wardrobe?
- R: Yes.
- C: Or on top of there, whichever one. Just put it there or something.
- R: We’ll just pop it up there, is that alright?
- C: Yes, that’s fine.

It is clear, then, that sensors come to be situated in specific locations with reference to the particular problems that occasion their introduction into the home; that there is a reflexive relationship between the articulation of problems, which warrant proposals being made to introduce sensing into the home, and the actual placement of sensors. A sensor is not, and cannot, be placed just anywhere. Rather, there is a close coupling between just where a sensor is placed and the reasons that accountably motivate its introduction. It is also plain to see that in the course of situating sensors advisors seek to understand clients’ problem management practices (e.g., constant wiping down). There is more to eliciting contextual data than simply filling in a form then, and there is more to situating sensors than coupling them to problems. Sensors are also placed to help the advisors understand the impact of domestic routines on the home and the client’s problem(s). Thus sensors are placed in locations that enable the advisors to understand occupancy patterns (through CO2 sensing), heating patterns (through temperature sensing) and the impact of routine activities such as cooking and bathing (through humidity sensing), etc.

The placement of multiple sensors around the home introduces a degree of complexity into the situating of sensors, as the following vignette elaborates:

- **Vignette 3. Checking the sensor kit**
- Checking the sensor kit
- A: Right, we’ll check now if the sensors are all working.
- R: XN in the kitchen is working. XD, upstairs bedroom, that one’s working. JE, above the room thermostat has not sent anything yet.
- A: OK. JD then is above the - on the room thermostat, I think. No, JD’s in the bedroom.
- R: No, XD’s in the upstairs bedroom.
- A: JD is the bedroom one but you’ve got it as XD.
- R: Oh, that’s why. So JD is there, that’s it then, that’s fine. XD is the outside one on the wall (…)
- A: Right.
- R: Great.
- A: So yes (turning to client), it shows the hub is all working and they’ve all sent readings in the last few minutes. So that’s fine, we know that it’s all working.

As the vignette makes visible, situating multiple sensors requires the advisor and researcher, and indeed anyone who might be doing this work, to “check” that the sensors are working. This is done by looking for “readings” on the hub, which turns not only on technical knowledge of sensor communications (e.g., waiting for a refresh) but also on the situated particulars of ‘just this’ installation. Thus, checking that the sensors are working also turns upon pairing readings with sensors (denominated by two-letter IDs) and sensors with locations, which as the above vignette shows is occasionally problematic. The problem is resolved by working through the mapping and matching sensor IDs to locations.^2^.

Once checks are completed and any issues resolved, the advisor returns to the business in hand and the projected future use of the data generated by the sensor kit:

- **Vignette 4. Projecting future use of the data**
- Projecting future use of the data
- A: Okay. So, do you know when the 26th - is it Tuesday?
- R: Yes, Tuesday.
- A: Whether you might be available in the afternoon, then we can pop back?
- C: Yes.
- A: It’s sending all the readings now so we can look at the data, particularly around the mould issue.
- C: Yes.
- A: We’ll offer some advice.
- C: Yes.
- A: Then we’ll leave the sensors in for another week. So in about two weeks? Around the 26th of January?
- C: Yes.

Thus, having installed the sensor kit, and checked that it is working, the advisor and client make specific arrangements to “look at the data” and for the advisor to “offer some advice” to the client “particularly around the mould issue.” Eliciting contextual data is more than a matter of simply completing a questionnaire then. When we look to see *what’s done in the doing* (Crabtree et al. 2012) of filling in the questionnaire, we see that the articulation of contextual data warrants the doing of a technical job of work that methodically provides a) for the introduction of IoT technology into the home, b) for situating it in particular locations with reference and respect to specific problems, and c) for the future use of data generated by the sensing kit to address those problems. We note too, that the improvements made to the contextual data capture instrument are indicative of an effort towards more systematic elicitation of contextual data or *metadata*. Metadata provides crucial information on the indexical relationship of sensor data to the sites and practices of its production. Without metadata then, it would be very difficult for advisors to make sense of the data, or to use it in a meaningful way within the subsequent provision of energy-related advice.

### Rehearsal

The next stage of data work centres on ‘rehearsing’ the collected sensor data in preparation for the advice visit. Rehearsal involves reading through the data to identify distinct patterns, which is done methodically in searching for ‘peaks and troughs’ in the data; speculating on the causes of these observable phenomenon, which draws on technical knowledge (e.g., of normal and abnormal heating cycles), local knowledge furnished through collection of contextual data, and common-sense knowledge; and annotating these data for discussion and verification with the client (Fischer et al. 2016). Three advisors took part in a workshop to work through the collected data and prepare themselves for the advice visits. The workshop served as a further site for observing how IoT sensor data is articulated and made sense of. The same basic jobs of work apply as reported in our previous study (ibid.) – i.e., identifying patterns, leveraging different bodies of knowledge to speculate on their causes, and annotating the data in preparation for the advice visit – *but* they are now accompanied by new methods for working with complex, multi-sensor datasets; methods that initially revolve around “getting an idea” of what a complex dataset produced by multiple sensors might be telling them:

- **Vignette 5. Getting an idea: identifying remarkable patterns**
- A: So what I would do first is to just look at the temperature data to begin with. So just to try and simplify first of all so we get an idea. [Filters out temperature data].
- A: Bloody hell, there’s some big fluctuations there. So the blue one at the bottom is the external [temperature]. Outside it goes down to nearly 0 up to 10. Inside some of the rooms are actually going down - above the room thermostat is going down - to 10 degrees. 10, 11 degrees is the lowest temperature. That’s actually quite low.
- A: So then what I do, I make some rough notes from that [temperature data] - if there are any particular periods to have a look at, or any peaks or troughs that need a bit further investigation. Then I look at the humidity. This is me just trying to find a way to kind of work through things really. So the external humidity, 70 up to maybe 90 the whole time over the last few weeks. In the different rooms it’s quite variable. Overall it’s not steady - problematic humidity given for all of the rooms, they are quite fluctuating; of all the rooms the upstairs bedroom is the most humid.

The vignette makes it visible that the advisors first need to “simplify” multi-sensor datasets to identify remarkable patterns, such as temperature or humidity fluctuations, and thus “get an idea” of what the problems are in a particular home. The simplification is done methodically by filtering out the noise created by multiple sensor feeds, focusing down on single data sources, and noting down remarkable occurrences (e.g., that the temperature is quite low, or that humidity is variable and problematic).

The advisors may then turn towards “correlating” multiple data sources to further elaborate energy-related patterns in the home:

- **Vignette 6. Correlating multiple data sources**
- A: In the lounge they’ve got the humidity, temperature and CO2 showing. They might have been away then because the CO2 is fairly quiet, it’s almost stable just there; not a lot happening. So there, the CO2 goes up. There. Temperature up there. So you’ve got roughly CO2 peaking with the temperature increases and it’s - just put in the gas use as well now, and turn off the humidity for a moment. Just trying to see if the CO2 and temperature coincide with gas use in terms of the heating, which - so yes, there’s a gas increase slightly behind the temperature increase. So that, yes, seems to correlate. And that one there where you’ve got the temperature staying high and then the CO2 is remaining quite high for a while.
- R: And that’s yes, sort of evening time, isn’t it?
- A: Yes.

As this vignette makes visible, the correlation of multiple data sources enables advisors to infer particular patterns. That, for example, the inhabitants of the home have “been away” from home because CO2 readings are “stable” and it’s plain to see that there’s “not a lot happening”. Conversely, correlating multiple data sources enables advisors to infer that and when people are at home as the reading starts to peak alongside a “temperature increase”, an occupancy pattern that is confirmed by “putting in the gas” to see if it “coincides” with CO2 and temperature, which it does. Taken together, single data sources enable the advisors to identify particular classes of problem in the home, and multiple data sources allow them to infer the patterns of human action that are implicated in their production. Thus, and for example, the correlation of multiple data sources enables an advisor to identify issues implicated in remarkable patterns seen through a single data source (such as occupancy and temperature fluctuations).

A further methodical feature of data work is found in the way that advisors earmark remarkable patterns and associated data for discussion in the advice visit. This was one of the main practices we sought to support digitally by means of the interactive system. However, this has turned out to involve not just the interactive system, but additional notes on paper, as this advisor explains to a colleague.

**Figure 3:**
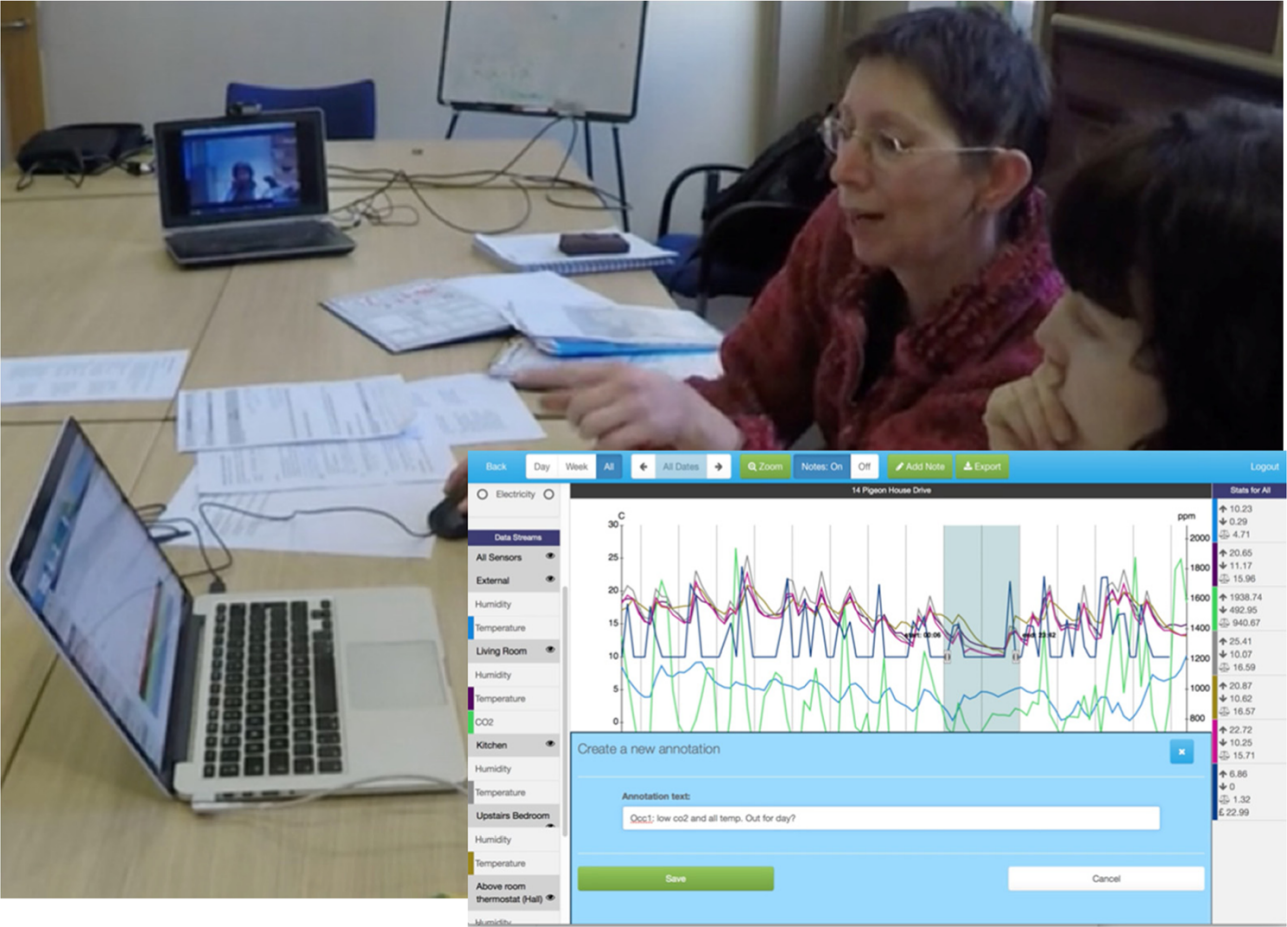
Advisor explaining ‘notation system’ to colleague during the rehearsal workshop (vignette 7a), and inset: added annotation (vignette 7b)

- **Vignette 7a. ‘Noting’ remarkable patterns**
- A: So, I’d probably go about putting in a note there (pointing at screen, Figure 3), annotating it as an area to explore with them [the client]. And I would try to use a kind of notation system, writing down an actual note on paper with the time and roughly what I would say. Just so I know where to go back on here (pointing at screen) - an indication, like as simply as possible.

‘Noting’ remarkable patterns and associated data is done methodically through the use of an ad hoc ‘notation system’. Thus the advisors have come to exploit the interactive system alongside paper, which is used to index or signpost the particular bits of data they want to discuss with the client. The notation system thus helps them locate relevant sections of data in the digital system. When prompted to elaborate this ‘notation system’, the advisor did so by example:

- **Vignette 7b. ‘Noting’ remarkable patterns (cont’d)**
- A: On this one [points at laptop] - we’re looking at Saturday 16^th^ to Monday the 18^th^ - I’m writing down the actual times of the period I’ve selected on the screen, and I’m going to call it an Occupancy [writes note on piece of paper]. If I think there’s going to be more than one, I’d give them a number. So I’m calling this ‘occupancy one’, just to try and make it simple. [Puts pen down and adds an annotation in the interactive system - **Occ1: low C02 and all temp. Out for the day?** (see Figure 3 inset)] So yes, low CO2, and all temperatures. And so what I do is I write on my home visit sheet [i.e., the piece of paper] what I’ve selected - so all temp and CO2 - so I know what to select to show the person: were they away? So now I know there’s something there [in the data].

The notation system involves both digital and physical annotations. The former is provided for through the interactive system. The latter is provided for through a bespoke accountability system: the “home visit sheet”. The sheet not only indexes the digital annotation, making it easy to locate specific parts of the dataset during an in-home visit and associated queries, it also lays out and defines an order of business to be addressed during the home visit (occupancy one, two, three, and so on). Thus, the home visit sheet is a coordinating device used methodically to order interaction between advisor and client. It provides a situationally-specific schedule of work that parses the overall dataset produced by the sensing kit and surfaces particular issues that need to be worked through with the client.

The advisors call the parsed dataset “reference data”. Reference data is data extracted from the overall dataset that the advisors deem to be relevant to understanding and addressing the client’s problem in some way, whether it be identifying remarkable patterns that articulate particular problems, such as temperature and humidity fluctuations, or raising queries about data that stand in need of clarification, such as the causes of low C02 and temperature data. The preparation of reference data for the advice visit involves the use of a “checklist”:

- **Vignette 8. Assembling reference data**
- A: I looked at the data yesterday and, using [the] checklist, I just went through some things with the data. Checking the maximum and minimum temperature recordings, looking at the differences in rooms, the variations between rooms, any sort of fluctuations within rooms, temperature patterns, anything sort of out of the ordinary.

The “checklist” formalises a professional practice for looking at the data, orienting advisors to “anything out of the ordinary” - maximum and minimum data points, differences, variations, fluctuations in and between rooms, etc. Finding “anything out of the ordinary” turns upon the various orders of knowledge and reasoning that the advisors possess and exploit in looking at and reading the data.

It is in the interplay between looking and reading that the advisors come to find things that are out of the ordinary and in turn, as the following vignette makes visible, formulate potential solutions to particular problems:

- **Vignette 9. Formulating potential advice**
- A: (…) relating it back to the initial inquiry, which was that her home was colder than she’d like in winter, she sometimes struggles with her energy bills. I’ve got some sort of tips on using her heating system more efficiently, like making use of the room thermostat. I don’t think she does at the moment. Possibly turning down the heating in the back bedroom, because that’s often above 21. Like it goes up to 25 on quite a few occasions. I don’t know if it’s just because it’s a small room.

Formulating potential advice turns upon sensor data (e.g., temperature readings), technical knowledge (e.g., of normal and abnormal heating cycles), common-sense knowledge (e.g., that abnormalities could, in this case, be caused by small room size), and contextual knowledge (e.g., of the client’s problems and practices). The combination of sensor data with these different orders of knowledge and reasoning enables advisors to go beyond offering general advice and provide situationally specific advice instead. Thus, and for example, an advisor can offer a client “tips” on using the heating system more efficiently such as “making use of the room thermostat”. Such fine-grained tips are provisional, however. They may resolve the client’s problems, but whether they do so or not has yet to be ratified.

To sum up, the ‘rehearsal’ stage of data work involves articulating problems and their potential solution. This is done by simplifying the dataset to identify ‘remarkable’ patterns in the data and correlating data sources to identify issues potentially implicated in their production. The work turns upon the use of technical, common-sense and contextual knowledge and the design and use of methods for ‘noting’ remarkable patterns and assembling ‘reference data’. Reference data parses the overall dataset and surfaces ‘anything out of the ordinary’, which enables advisors to formulate ‘tips’ that may resolve the problem situation. Reference data is indexed through the production of a home visit sheet, which allows advisors to quickly locate relevant data and defines a situationally-specific schedule of work ordering subsequent interaction between advisor and client in the performance of data work.

### Performance

The final stage of data work is methodically occupied with the ‘performance’ of data during the advice visit. This involves advisor and client articulating the remarkable patterns identified during rehearsal and pre-visit speculations as to their causes. The work here remains the same as detailed in (Fischer et al. 2016) and sees advisor and client articulating the relationship of data to problems, and tying problems to the client’s activities, practices and routines. In this way advisor and client verify or respecify pre-visit speculations, shape solutions around domestic priorities, and articulate future energy-related practices and their benefits. While our prior studies have shown how simple line charts are drawn upon as a collaborative resource supporting tailored advice-giving, data work now revolves around the interactive system and the articulation of multiple data sources earmarked on the home visit sheet. The following extract, drawn from the advice visit to the home with damp and mould problems encountered in vignette 1, provides an exemplar of the ways in which multiple sensor data is drawn upon to articulate problems and solutions.

- **Vignette 10. Articulating problems and solutions with reference to multiple sensors**
- A: Let’s just have a look at the temperature to begin. You see this is - all the different colours? So the purple one is the living room, and then the grey one the kitchen. The kind of olive coloured one is the upstairs bedroom (…)
- C: Yes.
- A: and in the hall is the pink one. So you can see they’re all quite similar in terms of the pattern (…)
- C: Yes.
- A: which is good. So you’re just popping your heating on then it’s just going through the house, which is great. It’s not like one room is particularly colder than the other. If we look at the - this shows, on this side, the average temperatures. The upstairs bedroom is the room with the mould, isn’t it?
- C: Yes.
- A: So we can see that on average, the highest temperature is about 20, the lowest is just above 11, and the average is 16 (…)
- C: Yes.
- A: In general the World Health Organisation recommends about 18 to 21 for health. So really you need to heat a bit more. What it’s showing is that the temperature is at 16, humidity [inaudible] high. You’ve got the condensation and mould up there, so increasing the temperature a little bit is going to kind of help to decrease that.
- C: Yes.
- A: I appreciate like maybe you haven’t got the money to heat a bit more, but we can maybe have a look at things that might be able to help you.

This vignette makes visible the methodical way in which advisors go about articulating a complex dataset produced by multiple sensors. Just as in rehearsal they filter the dataset to focus down on a single data source “to begin” with, though this time the filtering is driven by items earmarked on the home visit sheet. In this particular case the data source is related to the primary problem that affects the client’s home: temperature, which is indicative of potential damp and mould problems. The vignette also makes it visible how in articulating the data from a single source advisors explain what the data visualisation “shows”, thus offering an orienting description – “the purple one is the living room, and the grey one the kitchen… olive one the upstairs bedroom,” etc.

The data allows the advisor to articulate particular patterns (e.g., average temperatures throughout the home), and in turn to assess these (e.g., that they are “good”), and to relate them to normal expectations (as prescribed, for example, by general health guidelines). Visible discrepancies between the two enables the advisor to propose potential remedial action (e.g., “to heat a bit more”). Multiple data sources are introduced into the interaction to drill down into the problem (e.g., humidity in the upstairs bedroom) and drive home the advice: temperature is low, humidity high, so increasing the temperature is going to decrease the humidity levels. While multiple data sources enable deeper articulation of the client’s problems and their causes, this vignette also makes it plain to see that the advice they enable may also be problematic: e.g., increasing the heating in a low-income household.

That is not to say that nothing can be done about a client’s problem and advisors routinely go beyond data work to work through potential solutions (Fischer et al. 2016). In this particular case, the advisor and client left the data behind and went to inspect the damp and mould in the upstairs bedroom. In doing so the advisor articulated a range of options for managing the problem, including frequent wiping down, leaving the window ajar to increase ventilation, opening the curtains to let the sun warm the room, putting a reflective panel behind the radiator to increase heating efficiency, having the landlord check the roof insulation above the problem area, and turning the heating off when no one is in the house (a pattern also made visible by multiple data sources, particularly temperature, gas and CO2).

Overall, what is evident in the fieldwork observations is that the articulation of complex multi-sensor datasets frames and guides advice giving in important respects. In order to deal with the complexity of these datasets, advisors filter down to single data sources related to items earmarked for discussion and in doing so reflexively articulate and explain what the data is about and what it shows the client. Perceived problems are initially articulated with reference to a single data source, with multiple data sources being subsequently drawn upon to drill down into and elaborate problems. While client’s problems are often obvious, in that they already know what they are, multi-sensor datasets allow advisors to understand and articulate their causes (e.g., that low temperatures and high humidity are at the root of a particular damp and mould problem) and offer tailored advice to remedy the situation. The tailoring of advice is done with respect to the client’s circumstances and may also be informed by multi-sensor datasets, which is to say that such data is not only drawn on to articulate problems but also to articulate viable local solutions.

### Reflective workshop

After the sensor kit had been collected from clients’ homes, we conducted a reflective workshop with the advisors to understand their perspectives on the CharIoT system, how it fared within their work, and which features they found most supportive and useful for facilitating energy advice. When discussing the feature set of the interactive system, the advisors emphasised how they had used the system to highlight critical issues relating to *health*. As one advisor put it,

“ … flagging issues - being able to look at these graphs and immediately identify, you know, where homes are being under heated, and therefore potentially causing a health hazard.”

The advisors found value in the interactive system and appreciated the ability it gave them to “immediately identify” and “flag” potentially problematic issues*.*

The advisors also appreciated the use of multiple sensors, as this enabled them to compare rooms to one another and drill down into problems:

“The fact that we’ve got y’ know a number of sensors around the home rather than just one has been really beneficial as well. So some rooms are heated to 15 degrees and others are at a regular temperature. That room that’s heated to 15 degrees, in one instance that’s flagged repairs, things that need to be fixed, broken thermostatic radiator valves; or they’ve flagged you know just poor control where as a result of not heating that room to the temperature that it needs to be, you’ve got, you know, damp and mould issues.”

The use of multiple sensors has been “beneficial” to professional practice, enabling the advisors to both identify material problems with the home’s infrastructure (e.g., things that need to be fixed) as well as issues related to the use of that infrastructure (e.g., poor temperature control).

The advisors furthermore pointed out that the process of collecting and using sensor data made their role as experts more credible to their clients, and increased their clients’ engagement with the business of giving energy-related advice:

“ … being able to present that to householders and show them the relationship helps them engage with the issues … In previous visits people don’t necessarily believe what you’re saying, so being able to point that out …”

The perception that the sensor kit and interactive system helped clients engage with issues and “believe” what the advisors were saying underscores findings from our previous work, which suggests that IoT data can and does play a constructive role in building a trusting relationship between advisor and client.^3^.

## So what?

The perhaps more obvious takeaways from our work revolve around how design might support the organising features of anticipation, rehearsal, and performance of data work elaborated by *just this* study. For example, our insights on the production and use of annotations may inform the design of systems aiming to support similar practice. However, we would suggest that the methodological features of data work may be important to attend to *more generally* when designing collaborative, sociotechnical IoT systems. Our vignettes demonstrated that collaboration runs through all of the phases of data work. We are saying then, that the methodological matters we elaborate here are collaborative matters *through and through*. In the following, we discuss the ways in which these methodological matters speak to the ‘overhead cost’ of building collaboration into the IoT, which is to say that one cannot simply install a bunch of sensors, collect and process the data, and produce a situationally relevant and actionable answer.

Instead, our study makes it perspicuous in methodological detail that it is necessary to support the *social and collaborative nature* of sensor deployment, data analysis, and use to succeed in data work (situationally relevant and actionable energy advice in the case to hand). In this section, we develop this argument further by drawing on the CSCW literature on ‘overhead cost’, ‘appropriation’, and ‘workflow’. In doing so, we elaborate the social, ***collaborative methods of data work***. These methods sensitise those who seek to build and deploy IoT solutions to deliver personalised services across a number of key areas (from accounting for data capture to making data actionable).

Firstly though, how do we get from the specific to the general? How can any lessons or insights be drawn from our work to inform IoT development more generally? We have said before that we would suggest that in the first instance our study provides a ‘perspicuous setting’ (Crabtree et al. 2012), which instructs or teaches us about the issues involved in addressing a general problem; the general problem in this case being designing the IoT so it can be incorporated into and add value to everyday practice. Thus, the CSE study enables us to *learn from appropriation*. As Dix (2007) puts it,

“By observing the ways in which technology has been appropriated, we may then redesign the technology to better support the newly discovered uses. This is a form of co-design where the users are considered an integral part of the design process. This closing of the *Technology Appropriation Cycle* has been called *design from appropriation*.”

When we look at the work involved in incorporating the IoT into energy advice practice we can see that appropriation turns in significant ways upon what one advisor called “working through a method”, i.e., developing new working practices that methodically incorporate the IoT into existing working practices and thereby ‘domesticate’ (ibid.) the technology and ‘make it at home’ (Crabtree et al. 2012) in their world. The methods of which the advisor speaks are not formal methods devised and prescribed by an organisation but *members’ methods*; ad hoc methods devised by people to organise and conduct their activities: methods for introducing, situating, making sense of and making IoT data actionable, for example. We would suggest that the first take-away for design then lies in recognising that *method* is key to the *appropriation* of the IoT.

When we look to see what method consists of from the point of view of appropriation our study makes it perspicuous that it articulates discrete *workflows* (e.g., ‘anticipating’, ‘rehearsing’ and ‘performing’ data work), whose collaborative accomplishment makes the technology into a *routine* feature of everyday life. The methodical construction of discrete workflows means that users of the technology do not have to figure out use each and every time they encounter it. That they don’t have to reimagine what the technology could be used for and how. Rather, and to borrow from Gerson and Star (1986), it means that they have figured out how to ‘package’ the technology so as to ‘get the job done’ in the face of local contingencies (the particular home, the particular problems, the particular data produced, etc.) and thus come to incorporate the IoT into everyday practice.

Workflow is of course an established topic in CSCW, and one perhaps most effectively treated by Paul Dourish (2001). Dourish was curious as to why workflow systems had been so widely adopted by industry despite their evident failings, notably their overly prescriptive character and inflexibility in use, both of which negatively impact the performance of collaborative work. Dourish suggested that what explains this is the ‘dual role’ workflow systems play. That they are not only used to order and coordinate work, but to account for its performance to parties *external* to the work (e.g., customers) as well. Dourish thus suggests that workflow systems are ‘technologies of accountability’, which may be enhanced by ‘decoupling’ representations that account for work from ways and means of ordering it.

Without criticising Dourish’s original and imaginative take on workflow, our study also instructs us that accountability is an *internal* feature of workflow. As such, the advisors are methodically engaged in constructing technologies of accountability that allow them to incorporate the IoT into collaborative practice. Thus we have seen how the advisors have implemented a contextual survey to help them anticipate the demands of data work and furnish the metadata needed to conduct it, for example, and we have seen how they take notes on the home visit sheet in rehearsing the data. Both serve to make the placement of sensors and the data they generate accountable; and they provide resources that advisors and clients can collaboratively draw on to order the situationally relevant performance of data work. Our study thereby instructs us that the construction of discrete workflows turns upon the construction of *accountability systems* that enable the technology to be methodically incorporated in collaborative action.

Of course, neither the contextual survey nor the home visit sheet is digital. The latter draws on digital data, the former anticipates its production. This is not to say that they could not be digitally enabled and enacted. The point we are making in saying this is not so glib as to suggest that IoT development should completely digitise the end-to-end processes of data work (previous work has demonstrated the folly of such an approach, e.g., Bowers et al. 1995). Rather, we are saying that IoT development should ‘take CSCW seriously’ (Schmidt and Bannon 1992) and recognise the *overhead cost of collaboration*. IoT development might benefit, then, by facilitating discrete workflows and accountability systems, especially where automated IoT systems are concerned, such as automated energy-advice systems that take the advisor out of the loop. Only a few of the 2.38 million homes in the UK that are affected by fuel poverty will receive face-to-face advice. It is more likely that they will be confronted by IoT systems that are installed by energy providers, housing associations, landlords, etc. In order to add real value these too will need to support the methodical accomplishment of collaborative action, as this demonstrably enables the technology’s *appropriation* in everyday practice. It might otherwise be said, for clarity’s sake, that the methodical accomplishment of collaborative action constitutes ‘the work to make the technology work’, which, as CSCW researchers have previously pointed out is key to the efficacy of automated systems (e.g., Bowers 1994; Grinter et al. 2005).

In turn, the takeaways from our study we wish to highlight are the ***collaborative methods of data work*** in and through which accountability in data work is achieved***.*** We would suggest that it may be important to take these methods into account when designing for IoT systems as sociotechnical systems, including accounting for data capture and use, situating sensors, capturing contextual metadata, making sense of the data, and turning data into action:

- ***Methods of accounting for data capture and use*** *.* At the point of introducing IoT systems into real world settings (or potentially exploiting sensing that is already in situ), it is important to make data capture and use accountably reasonable (see vignettes 1 and 4, for example). The aim here is to make the purposes of data capture (what the data will be used for) and mechanisms of data capture (including data transmission, storage and access) transparent *and* legible to users of the system. Accountability also turns upon developing mechanisms that provide for informed consent and enable data privacy, and these are particularly important matters to consider in automated systems.^4^
- ***Methods supporting the situating of sensors.*** Situating sensors (or potentially exploiting sensors that are already situated), is a purposeful activity done with respect to particular problems and/or goals, which need to be worked out appropriately with those living with the sensors (see vignettes 2 and 3, for example**)**. Mechanisms need to be designed, particularly in automated systems, that take account of the specifics of the environment into which sensors are placed and used (e.g., just where they are situated), the technical capabilities and constraints of particular sensing platforms (e.g., sensor type, reach, and limitations), and ensure that the technology works correctly and captures data related to the problem/goal in an acceptable (e.g., non-intrusive) way.
- ***Methods for capturing contextual metadata.*** Contextual metadata is required to understand and unpack the indexical relationship of sensor data to the sites and practices of its production (see vignette 1, for example). Metadata not only includes mapping sensors and capturing the location of their real-world placements, but situationally-relevant information that is needed to make sense of data outputs (e.g., metadata about the human activities, practices and routines implicated in their production). In automated systems, mechanisms enabling those present in the space to articulate relevant contextual metadata will need to be built-in to enable effective future use of the data.
- ***Methods for making sense of the data.*** Making effective use of the data turns on upon parsing complex multi-sensor data sets to identify discrete patterns that are clearly related to the purposes for which sensing is being done (see vignettes 5 to 9, for example). Where the goal is to address particular problems and issues that are (potentially) implicated in their production, it will be necessary to develop mechanisms that ‘flag’ remarkable patterns and surface ‘anything out of the ordinary’. It may also be necessary to built-in collaborative feedback loops to enable end-users to add further metadata to verify or respecify understandings of the data.
- ***Methods for turning data into action.*** Taking action turns not just upon identifying but also negotiating the potential courses of action that might be taken in response to the relevant patterns in the data **(**see vignette 10, for example**)**. Methods of negotiation turn on anticipating the potential impact of projected beneficial effects on an individual’s or cohort’s circumstances (e.g., on their finances). Potential actions may be contested and are contingent to the situated circumstances and practices into which they must fit.

## Conclusions

This paper has presented fieldwork findings from deploying the CharIoT system used by advisors working for a charity to support the provision of energy-related advice to households. Our findings document how energy advisors and their clients articulate the system and the data it produced, and how they collaboratively anticipate, rehearse, and perform *data work*. The study presented here concludes two years of research, spanning sensitising ethnographic work (Fischer et al. 2014); initial prototyping and deployment of a single sensing device (Fischer et al. 2016); and, in turn, the co-design of the CharIoT system featuring multiple sensing devices and an interactive web app. This paper examines deployments of the CharIoT system, thus providing further insight into the work required to perform data work in more complex data rich ecosystems. Our findings detail how the multi-sensor system was appropriated by the advisors and employed to deliver tailored advice to their clients.

The paper contributes to IoT development more generally, with the study elaborating the ‘overhead cost’ of building collaborative action into connected devices and sensing systems. In this respect, the study elaborates how the appropriation of IoT technologies turns upon members’ methods, which provide for and articulate discrete workflows and accountability systems enabling sensing systems to be incorporated into collaborative action. While it is a contingent matter as to just what workflows and concomitant accountability systems will need to be built into sensing systems more generally, we suggest that several ***collaborative methods of data work*** may be important to attend to when factoring in the overhead cost of collaboration to the IoT. These include accounting for data capture and use, situating sensors, capturing contextual metadata, making sense of the data produced through the IoT, and turning this data into action. Our intention in this work was to take a sociotechnical CSCW lens to IoT systems design to emphasise collaboration as a major concern within it.

## Footnotes

1 The CharIoT system is available open source at https://github.com/horizon-institute/chariot with instructions on how to set up a web server, configure the sensors, and the hub.

2 This is a simple task in the current situation, but not necessarily so in a foreseeable future in which widespread energy-related advice is provided by automated external IoT services that exploit self-installed sensors. ‘Checking’ may then become a discrete job of collaborative work that requires explicit support.

3 Perceived benefits of the interactive IoT system are further elaborated by members of the Centre for Sustainable Energy here: https://www.youtube.com/watch?v=jcDbR9YVY6k

4 Recent work that rethinks consent models and data privacy provides innovative exemplars (Luger and Rodden 2013; Chaudhry et al. 2015; Crabtree et al. 2016).

## References

[CR1] Bannon, Liam; John Bowers; Peter Carstensen; John A. Hughes; Kari Kuutii; James Pycock; Tom Rodden; Kjeld Schmidt; Dan Shapiro; Wes Sharrock; and Stephen Viller (1993). *Informing CSCW System Requirements*, COMIC Deliverable 2.1. Lancaster University, United Kingdom: Computing Department.

[CR2] Bedwell, Ben D.; Enrico Costanza; and Michael Jewell (2016). Understanding Energy Consumption at Work: Learning from Arrow Hill. In *CSCW ‘16*: *Proceedings of the 19th ACM Conference on Computer-Supported Cooperative Work & Social Computing, San Francisco, USA, February 27–March 2, 2016.* New York: ACM Press, pp. 1335–1346.

[CR3] Bowers, John (1994). The work to make a network work: studying CSCW in action. In *CSCW**‘94: **Proceedings of the 1994 ACM conference on Computer supported cooperative work, Chapel Hill, North Carolina, USA, October 22 - 26, 1994.* New York: ACM Press, pp. 287–298.

[CR4] Bowers J, Button G, Sharrock W, Marmolin H, Sundblad Y, Schmidt K (1995). Workflow from within and without: technology and cooperative work on the print industry shopfloor. *ECSCW ‘95: Proceedings of the Fourth European Conference on Computer-Supported Cooperative Work, Stockholm, Sweden, 11 - 15 September, 1995*.

[CR5] Button G, Harper R (1995). The Relevance of “Work-practice” for Design. Computer Supported Cooperative Work (CSCW).

[CR6] Button, Graham; Andy Crabtree; Mark Rouncefield; and Peter Tolmie (2015). Deconstructing Ethnography. London: Springer.

[CR7] Chaudhry, Amir; Jon Crowcroft; Heidi Howard; Anil Madhavapeddy; Richard Mortier; Hamed Haddadi, and Derek McAuley (2015). Personal data: thinking inside the box. In *AA ‘15: Proceedings of The Fifth Decennial Aarhus Conference on Critical Alternatives*, *Aarhus, Denmark, August 17 - 21,* 2015, New York: ACM Press, pp. 29–32.

[CR8] Chetty, Marshini; Richard Banks; Richard Harper; Tim Regan; Abigail Sellen; Christos Gkantsidis; Thomas Karagiannis; and Peter Key (2010). Who’s hogging the bandwidth: the consequences of revealing the invisible in the home. In *CHI ‘10: Proceedings of the 28th international conference on Human factors in computing systems, Atlanta, Georgia, USA, April 10 - 15, 2010*. New York: ACM Press, pp. 659–668.

[CR9] Costanza, Enrico; Sarvapali D. Ramchurn; and Nicholas R. Jennings (2012). Understanding domestic energy consumption through interactive visualisation. In *UbiComp ‘12: Proceedings of the 2012 ACM Conference on Ubiquitous Computing*, *Pittsburgh, Pennsylvania, September 05 - 08, 2012*. New York: ACM Press, pp. 216–225.

[CR10] Crabtree A, Lodge T, Colley J, Greenhalgh C, Mortier R, Haddadi H (2016). Enabling the new economic actor: data protection, the digital economy, and the Databox. Personal and Ubiquitous Computing.

[CR11] Crabtree, Andy; and Richard Mortier (2015). Human Data Interaction: Historical Lessons from Social Studies and CSCW. In N. Boulus-Rødje; G. Ellingsen, T. Bratteteig; M. Aanestad; and P. Bjorn (eds.): *ECSCW ‘15: Proceedings of the 14th European Conference on Computer Supported Cooperative Work*, *Oslo, Norway, 19-23 September 2015.* Cham: Springer, pp. 3–21.

[CR12] Crabtree, Andrew; Tom Rodden; Peter Tolmie; and Graham Button (2009). Ethnography Considered Harmful. In *CHI ‘09: Proceedings of the SIGCHI Conference on Human Factors in Computing Systems*, *Boston, MA, USA, April 04 - 09, 2009.* New York: ACM Press, pp. 879–888.

[CR13] Crabtree A, Rouncefield M, Tolmie P (2012). *Doing Design Ethnography*.

[CR14] DECC (2016) *Annual Fuel Poverty Statistics Report, 2016*. Department of Energy and Climate Change.

[CR15] Dillahunt, Tawanna R.; and Jennifer Mankoff (2014). Understanding factors of successful engagement around energy consumption between and among households. In *CSCW ‘14*: *Proceedings of the 17th ACM conference on Computer supported cooperative work & social computing, Baltimore, Maryland, USA, February 15 - 19, 2014.* New York: ACM Press, pp. 1246–1257.

[CR16] Dillahunt, Tawanna; Jennifer Mankoff; and Eric Paulos (2010). Understanding conflict between landlords and tenants. In *Ubicomp ‘10: Proceedings of the 12th ACM international conference on Ubiquitous computing*, *Copenhagen, Denmark, September 26 - 29, 2010.* New York: ACM Press, pp. 149–158.

[CR17] Dillahunt, Tawanna; Jennifer Mankoff; Eric Paulos; and Susan Fussell (2009). It’s not all about “Green”: energy use in low-income communities. In *Ubicomp ‘09*: *Proceedings of the 11th international conference on Ubiquitous computing, Orlando, Florida, USA, September 30 - October 03, 2009.* New York: ACM Press, pp. 255–264.

[CR18] DiSalvo, Carl; Phoebe Sengers; and Hrönn Brynjarsdóttir (2010). Mapping the landscape of sustainable HCI. In *CHI ‘10: Proceedings of the 28th international conference on Human factors in computing systems*, *Atlanta, Georgia, USA, April 10 - 15, 2010*. New York: ACM Press, pp. 1975–1984.

[CR19] Dix, Alan (2007). Designing for Appropriation. In *Proceedings of the 21st British HCI Group Annual Conference on People and Computers: HCI...But Not As We Know It - Volume 2*, *University of Lancaster, United Kingdom, September 03 - 07, 2007*. British Computer Society, pp. 27–30.

[CR20] Dong, Tao; Mark W. Newman; Mark S. Ackerman; and Sarita Schoenebeck (2015). Supporting reflection through play: field testing the home trivia system. In *UbiComp ‘15: Proceedings of the 2015 ACM International Joint Conference on Pervasive and Ubiquitous Computing*, *Osaka, Japan — September 07 - 11, 2015.* New York: ACM Press, pp. 251–262.

[CR21] Dourish, Paul (2001). Process Descriptions As Organisational Accounting Devices: The Dual Use of Workflow Technologies. In *GROUP ‘01: Proceedings of the 2001 International ACM SIGGROUP Conference on Supporting Group Work*, *Boulder, Colorado, USA, September 30 - October 03, 2001*. New York: ACM Press, pp. 52–60.

[CR22] Dutton, William H.; and Grant Blank (2013). Cultures of the Internet: the Internet in Britain. *InterMedia,* vol. 42, no. 4/5, Winter 2014/15, pp. 55–57.

[CR23] Fischer, Joel E.; Enrico Costanza; Sarvapali D. Ramchurn; James Colley; and Tom Rodden (2014). Energy advisors at work: charity work practices to support people in fuel poverty. In *UbiComp ‘14: Proceedings of the 2014 ACM International Joint Conference on Pervasive and Ubiquitous Computing*, *Seattle, Washington, September 13 - 17, 2014.* New York: ACM Press, pp. 447–458.

[CR24] Fischer, Joel E.; Andy Crabtree; Tom Rodden; James A. Colley; Enrico Costanza; Michael O. Jewell; and Sarvapali D. Ramchurn (2016). “Just whack it on until it gets hot”: Working with IoT Data in the Home. In *CHI ‘16: Proceedings of the 2016 CHI Conference on Human Factors in Computing Systems*, *Santa Clara, California, USA, May 07 - 12, 2016.* New York: ACM Press, pp. 5933–5944.

[CR25] FTC staff (2015). *Internet of Things: Privacy and Security in a Connected World*. Technical Report. Federal Trade Commission.

[CR26] Garfinkel H (1967). *Studies in Ethnomethodology*.

[CR27] Garfinkel H, Button G (1991). Respecification: Evidence for locally produced, naturally accountable phenomena of order, logic, reason, meaning, method, etc. in and as of the essential haecceity of immortal ordinary society (I)—an announcement of studies. *Ethnomethodology and the Human Sciences*.

[CR28] Gerson EM, Star SL (1986). Analyzing due process in the workplace. ACM Transactions on Information Systems.

[CR29] Goecks, Jeremy; Amy Voida; Stephen Voida; and Elizabeth D. Mynatt (2008). Charitable technologies. In *CSCW ‘08: Proceedings of the ACM 2008 conference on Computer supported cooperative work, San Diego, CA, USA, November 08 - 12, 2008*. New York: ACM Press, pp. 689–698.

[CR30] Grinter RE, Keith Edwards W, Newman MW, Ducheneaut N, Gellersen H, Schmidt K, Beaudouin-Lafon M, Mackay W (2005). The work to make a home network work. *ECSCW ‘05: Proceedings of the Ninth European Conference on Computer-Supported Cooperative Work, 18-22 September 2005, Paris, France*.

[CR31] Jacobs, Rachel; Steve Benford; Mark Selby; Michael Golembewski; Dominic Price; and Gabriella Giannachi (2013). A conversation between trees: what data feels like in the forest. In *CHI ‘13: Proceedings of the SIGCHI Conference on Human Factors in Computing Systems*, *Paris, France, April 27 - May 02, 2013.* New York: ACM Press, pp. 129–128.

[CR32] Jiang, Yifei; Li Shang; Kun Li; Lei Tian; Ricardo Piedrahita; Xiang Yun; Omkar Mansata; Qin L; Robert P. Dick; and Michael Hannigan (2011). MAQS: a personalized mobile sensing system for indoor air quality monitoring. In *UbiComp ‘11: Proceedings of the 13th international conference on Ubiquitous computing*, *Beijing, China, September 17 - 21, 2011*. New York: ACM Press, pp. 271–280.

[CR33] Kim, Sunyoung; and Eric Paulos (2010). InAir: sharing indoor air quality measurements and visualizations. In *CHI ‘10*: *Proceedings of the 28th international conference on Human factors in computing systems, Orlando, Florida, USA, September 30 - October 03, 2009.* New York: ACM Press, pp. 1861–1870.

[CR34] Luger, Ewa; and Tom Rodden (2013). An informed view on consent for UbiComp. In *UbiComp ‘13: Proceedings of the 2013 ACM international joint conference on Pervasive and ubiquitous computing*, *Zurich, Switzerland, September 08 - 12, 2013.* New York: ACM Press, pp. 529–538.

[CR35] Merkel, Cecelia; Umer Farooq; Lu Xiao; Craig Ganoe; Mary Beth Rosson; and John M. Carroll (2007). Managing technology use and learning in nonprofit community organizations. In *CHIMIT ‘07: Proceedings of the 2007 symposium on Computer human interaction for the management of information technology, Cambridge, Massachusetts, March 30 - 31, 2007.* New York: ACM Press, no. 8.

[CR36] Merkel, Cecelia B.; Lu Xiao; Umer Farooq; Craig H. Ganoe; Roderick Lee; John M. Carroll; and Mary Beth Rosson (2004). Participatory design in community computing contexts. In *PDC ‘04: Proceedings of the eighth conference on Participatory design Artful integration: interweaving media, materials and practices*, *Toronto, Ontario, Canada, July 27 - 31, 2004*. New York: ACM Press, pp. 1–10.

[CR37] Pierce, James; and Eric Paulos (2012). Beyond energy monitors: interaction, energy, and emerging energy systems. In *CHI ‘12: Proceedings of the 2012 ACM annual conference on Human Factors in Computing Systems*, *Austin, Texas, USA, May 05 - 10, 2012*. New York: ACM Press, pp. 665–674.

[CR38] Pousman, Zachary; Mario Romero; Adam Smith; and Michael Mateas (2008). Living with tableau machine: a longitudinal investigation of a curious domestic intelligence. In *UbiComp ‘08*: *Proceedings of the 10th international conference on Ubiquitous computing, Seoul, Korea, September 21 - 24, 2008.* New York: ACM Press, pp. 370–379.

[CR39] Robertson T, Wagner I, Boulus-Rødje N, Ellingsen G, Bratteteig T, Aanestad M, Bjorn P (2015). CSCW and the Internet of Things. *ECSCW 2015*. *ECSCW ‘15: Proceedings of the 14th European Conference on Computer Supported Cooperative Work*, *Oslo, Norway, 19-23 September 2015*.

[CR40] Schmidt, Kjeld; and Liam Bannon (1992). Taking CSCW seriously: supporting articulation work. *Computer Supported Cooperative Work (CSCW),* vol. 1, nos. 1–2, pp. 7–40.

[CR41] Scott, James; A.J. Bernheim Brush; John Krumm; Brian Meyers; Michael Hazas; Stephen Hodges; and Nicolas Villar (2011). PreHeat: controlling home heating using occupancy prediction. In *UbiComp ‘11: Proceedings of the 13th international conference on Ubiquitous computing*, *Beijing, China, September 17 - 21, 2011*. New York: ACM Press, pp. 281–290.

[CR42] Stoll, Jennifer; W. Keith Edwards; and Elizabeth D. Mynatt (2010). Informal interactions in nonprofit networks. In *CHI ‘10*: *Proceedings of the 28th international conference on Human factors in computing systems, Orlando, Florida, USA, September 30 - October 03, 2009.* New York: ACM Press, pp. 533–536.

[CR43] Strauss A (1985). Work and the division of labor. Sociological Quarterly.

[CR44] Strengers, Yolande A.A. (2011). Designing eco-feedback systems for everyday life. In *CHI ‘11*: *Proceedings of the 2011 annual conference on Human factors in computing systems, Vancouver, BC, Canada, May 07 - 12, 2011*. New York: ACM Press, 2135–2144.

[CR45] Tolmie, Peter; and Andy Crabtree (2008). Deploying research technology in the home. In *CSCW ‘08: Proceedings of the ACM 2008 conference on Computer supported cooperative work, San Diego, CA, USA, November 08 - 12, 2008*. New York: ACM Press, pp. 639–648.

[CR46] Tolmie, Peter; Andy Crabtree; Tom Rodden; James A Colley; and Ewa A Luger (2016). “This has to be the cats” - Personal Data Legibility in Networked Sensing Systems. In *CSCW ‘16*: *Proceedings of the 19th ACM Conference on Computer-Supported Cooperative Work & Social Computing, San Francisco, USA, February 27–March 2, 2016.* New York: ACM Press, pp. 490–501.

[CR47] Voida, Amy; Ellie Harmon; and Ban Al-Ani (2012). Bridging between organizations and the public: volunteer coordinators’ uneasy relationship with social computing. In *CHI ‘12: Proceedings of the 2012 ACM annual conference on Human Factors in Computing Systems*, *Austin, Texas, USA, May 05 - 10, 2012*. New York: ACM Press, pp. 1967–1976.

[CR48] Walport M (2014). *The Internet of Things: making the most of the second digital revolution*.

[CR49] Zimmerman D, Pollner M, Pepinsky HB (1970). The everyday world as a phenomenon. *People and Information*.

